# Fabrication of porous silicon by metal-assisted etching using highly ordered gold nanoparticle arrays

**DOI:** 10.1186/1556-276X-7-450

**Published:** 2012-08-09

**Authors:** Sebastian P Scheeler, Simon Ullrich, Stefan Kudera, Claudia Pacholski

**Affiliations:** 1 Max Planck Institute for Intelligent Systems, Department of New Materials and Biosystems, Heisenbergstrasse 3, Stuttgart, 70569, Germany

**Keywords:** Porous silicon, Nanolithography, Gold nanoparticles, Self-assembly, Metal-assisted etching, 81.05.Rm, 81.16.Nd, 81.65.Cf

## Abstract

A simple method for the fabrication of porous silicon (Si) by metal-assisted etching was developed using gold nanoparticles as catalytic sites. The etching masks were prepared by spin-coating of colloidal gold nanoparticles onto Si. An appropriate functionalization of the gold nanoparticle surface prior to the deposition step enabled the formation of quasi-hexagonally ordered arrays by self-assembly which were translated into an array of pores by subsequent etching in HF solution containing H_2_O_2_. The quality of the pattern transfer depended on the chosen preparation conditions for the gold nanoparticle etching mask. The influence of the Si surface properties was investigated by using either hydrophilic or hydrophobic Si substrates resulting from piranha solution or HF treatment, respectively. The polymer-coated gold nanoparticles had to be thermally treated in order to provide a direct contact at the metal/Si interface which is required for the following metal-assisted etching. Plasma treatment as well as flame annealing was successfully applied. The best results were obtained for Si substrates which were flame annealed in order to remove the polymer matrix - independent of the substrate surface properties prior to spin-coating (hydrophilic or hydrophobic). The presented method opens up new resources for the fabrication of porous silicon by metal-assisted etching. Here, a vast variety of metal nanoparticles accessible by well-established wet-chemical synthesis can be employed for the fabrication of the etching masks.

## Background

Porous silicon has been extensively studied in the last 30 years due to its high potential for a variety of applications such as in optoelectronic devices [1], sensors [2], and solar cells [3,4]. Based on the first discovery of porous silicon formation by Uhlir at Bell Laboratories, the most popular method for porosification of silicon is anodic electrochemical dissolution in fluoride-containing solutions [5]. Extensive work has been conducted in order to understand the etching mechanism and to provide experimental parameters for the formation of porous silicon with controlled pore size, density, morphology, and depth [6]. In addition electroless etching of silicon, referred to as stain etching in an aqueous solution of HNO_3_ and HF, has been used for the fabrication of porous silicon [7]. However, the chemical reactions involved are extremely complex and, consequently, hard to control resulting frequently in thin as well as inhomogeneous porous silicon layers. Quite recently, Kolasinski and co-workers made considerable progress in chemical etching of silicon by replacing HNO_3_ with other oxidants such as Ce_4_^+^, Fe_3_^+^, and VO_2_^+^. These stain etching formulations provided homogenous porous silicon films [8]. A major limitation of stain etching is the range of accessible pore sizes. Here, pore diameters of up to only a few nanometers can be achieved, which is a narrow window compared to the nanometer to micrometer range accessible with anodic electrochemical etching. This drawback of chemical etching was compensated for by the discovery of metal-assisted etching which utilizes noble metal deposits on Si in order to increase its dissolution rate upon etching in a mixture of HF and an oxidative agent [9]. The chosen noble metal, as well as the morphology of the metal deposit, has a strong influence on the generated porous silicon. Consequently, a considerable amount of studies have been published demonstrating the advantages of this method [10,11]. It is not only simple and low-cost, but also allows for controlling the properties of the resulting nanostructure including cross-sectional shape, diameter, depth, and orientation of the pores. From this perspective it is surprising that metal deposition was almost exclusively achieved by techniques such as sputtering, electroless deposition, and thermal evaporation, leading to the formation of metal nanostructures with undefined shape, broad size distribution, and undefined interparticle spacing [12]. Only recently, lithographically fabricated metal structures were employed for metal-assisted etching [13,14]. The vast variety of metal nanoparticles with defined shapes and small size distribution provided by colloidal chemistry routes [15] was almost completely neglected. One reason might be the challenges in depositing a monolayer of metal nanoparticles on a surface with sufficient interparticle distances. Here, a method for the formation of a highly ordered gold nanoparticle array on Si is presented which can be translated into an array of ordered pores by metal-assisted etching. For this purpose gold nanoparticles were wet-chemically synthesized, furnished with polystyrene ligands, and spin-coated onto p-type Si. The self-assembled gold nanoparticle arrays were thermally treated in order to remove the polystyrene shell and subsequently etched in a solution composed of HF and H_2_O_2_.

## Methods

All reagents were used as received. Silicon wafer ((1 0 0), p-type, boron-doped, resistivity of 0.01 to 0.02 Ω cm) were purchased from Siegert Consulting e.K. (Siegert Wafer GmbH, Aachen, Germany). HAuCl_4_·3 × H_2_O, cetyltrimethylammonium bromide (CTAB), and sodium citrate were supplied by Sigma Aldrich (Sigma-Aldrich Chemie GmbH, Munich, Germany). Ethanol and H_2_SO_4_ were received from Carl Roth. H_2_O_2_ and toluene were obtained from Merck (Merck KGaA, Darmstadt, Germany). The l(+)-ascorbic acid and methanol were received from J. T. Baker (Mallinckrodt Baker B.V., Deventer, Netherland). Oleylamine was supplied by Acros (Acros Organics, Belgium, USA). HF was purchased from Merck. Thiol-terminated polystyrene (PS50:P4434-SSH, Mn = 50,000 gmol/1, Mn/Mw = 1.06) was obtained from Polymer Source Inc. (Quebec, Canada). Water was deionized to a resistance of at least 18.2 MΩ (Ultra pure water system (TKA Thermo Electron LED GmbH, Stockland, Niederelbert, Germany)) and then filtered through a 0.2-μm filter. Scanning electron microscope (SEM) images were taken with a Zeiss Ultra 55 ‘Gemini’ SEM (Carl Zeiss AG, Oberkochen, Germany).

### Fabrication method

**Figure 1:**
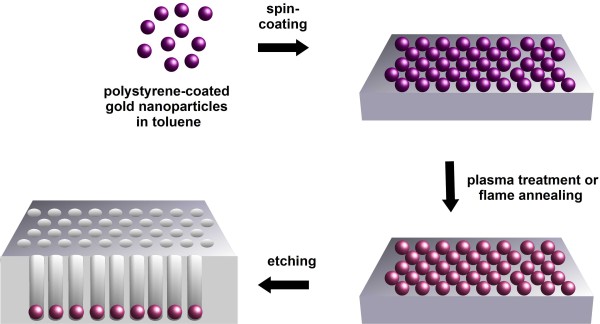
**Scheme of the fabrication method.** Polystyrene-coated gold nanoparticles were deposited on silicon substrates using spin-coating. The generated highly ordered arrays of gold nanoparticles were thermally treated in order to remove the polymer matrix. Finally, the substrates were etched in a mixture of HF and H_2_O_2_ resulting in the formation of porous silicon.

Figure 1 displays the fabrication steps of porous silicon by metal-assisted etching using highly ordered gold nanoparticle arrays as an etching mask. Briefly, spherical gold nanoparticles were synthesized in aqueous solution according to Maus et al. [16], transferred to toluene using oleylamine as ligand, and finally, covered with thiol-terminated polystyrene (SU, SS, CP, SK, and JP Spatz, unpublished work). The polymer shell enables the formation of highly ordered arrays of gold nanoparticles by self-assembly. The deposition of the nanoparticles was achieved by spin-coating. The interparticle distance of the gold nanoparticles was mainly determined by the employed polymer. As metal-assisted etching demands direct contact between metal and Si, the polymer matrix was removed using either plasma treatment or flame annealing. Finally, the silicon substrates were etched in a solution containing 4.8 M HF and 0.4 M H_2_O_2_. 

### Gold nanoparticle synthesis

Gold nanoparticles were synthesized using a seeded growth approach. First, gold nanoparticle seeds were prepared according to Frens [17]. Briefly, 39.4 mg (0.1 mmol) of gold(III)chloride trihydrate was dissolved in 100 mL deionized water. The solution was heated to boil, and 114 mg (0.4 mmol) of sodium citrate dihydrate dissolved in 10 mL deionized water was injected to the boiling solution under vigorous stirring. After 30 min reaction time, the resulting gold nanoparticle solution was cooled to room temperature. The solution was filtered using a 0.8-μm syringe filter prior to the subsequent growth reaction which was carried out according to Maus et al. Briefly 2,383 g (34 mmol) of hydroxylamine hydrochloride was dissolved in 3,550 mL deionized water to which 74 mL of the seed particle dispersion was added under stirring. Subsequently, 1,176 g (3 mmol) of gold(III) chloride trihydrate dissolved in 4,430 mL deionized water was slowly added to the solution with a constant flow of 420 mL/h. The solution was stirred for 8 h and after addition of 20 g of CTAB, was stirred for additional 3 h. Finally, the gold nanoparticle dispersion was centrifuged (15,000 × *g*, 15 min). The supernatant was discarded and the centrifugate was dispersed in deionized water. This procedure was repeated several times in order to remove the free CTAB. In addition the gold nanoparticle size distribution was narrowed by size-selective precipitation.

### Functionalization of the gold nanoparticles with polystyrene

The gold nanoparticles were covered with polystyrene using a method which has been recently developed in our group. Here, the gold nanoparticles centrifugate was dispersed in 20 mL oleylamine. After ultrasonication for 15 min, the dispersion was heated to 270°C for 1 h under stirring in an inert atmosphere. The thermal treatment removed the CTAB from the particle surface. After cooling to room temperature, the dispersion was centrifuged (15 min, 5,200 × *g*). The supernatant was discarded, and the particles were dispersed in a mixture of 5 mL oleylamine and 20 mL toluene. In order to cover the gold nanoparticles with polystyrene ligands, the oleylamine functionalized gold particles in toluene were precipitated by adding methanol. The particles were isolated by centrifugation, and 5 mL of polymer solution composed of thiol-terminated polystyrene and toluene (5 mg/1 mL) was added to the centrifugate. After ultrasonication for 15 min, the dispersion was incubated for 2 days. This process was repeated twice and resulted in polystyrene-functionalized gold nanoparticles dispersed in toluene. The overall yield of the preparation of polystyrene-covered gold nanoparticles with a diameter of 55 ± 9 nm is 17% in relation to the amount of used gold salt. Uniform particle monolayers on Si substrates were obtained after appropriate adjustment of the particle concentration.

### Spin-coating

Si substrates (p-type, boron doped, (100) orientation, resistivity of 0.01 to 0.02 Ω cm, size of 20 × 7 mm) were immersed in either piranha solution (concentration of H_2_SO_4_:H_2_O_2_, 3:1, *v/v*) for 1 h or in 4.8 M HF solution for at least 10 min prior to spin-coating. The substrates were afterwards rinsed with deionized H_2_O as well as with ethanol, in the case of HF treatment, and were blown dry with N_2_. Ten microliters of polystyrene-functionalized gold nanoparticles dispersed in toluene were spin-coated onto the substrates at 2,000 rpm for 1 min (spin coater, Laurell Technologies Corporation North Wales, PA, USA; Model WS-400B-6NPP/LITE).

### Polymer matrix removal

Polystyrene was removed from the gold nanoparticle surface by plasma treatment using a PVA TePla 1000 Plasma system (PVA TePla AG, Munich-Feldkirchen, Germany) (W10 (90% argon + 10% hydrogen), 150 W, 0.4 mbar, 45 min) or flame annealing. The latter was performed by pulling the substrate several times through a propane/butane flame.

### Metal-assisted etching

The substrates decorated with bare gold nanoparticles were etched in 4.8 M HF + 0.4 M H_2_O_2_ for different time periods. Afterwards, the substrates were thoroughly rinsed with ethanol and then dried under a stream of nitrogen.

## Results and discussion

**Figure 2:**
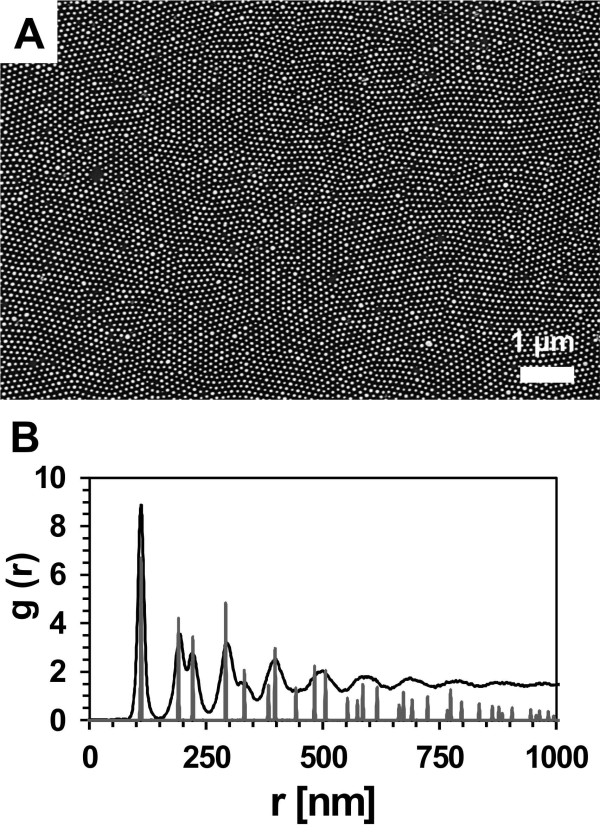
**Characterization of highly ordered gold nanoparticle arrays.** Gold nanoparticles were deposited onto silicon which has been cleaned with piranha solution. (**A**) SEM image of a highly ordered gold nanoparticle array. The diameter of the gold nanoparticles was 55 ± 9 nm and the interparticle distance was 111 ± 13 nm. (**B**) Corresponding pair distribution function.

Figure 2A shows a representative SEM image of a gold nanoparticle array on Si. In order to demonstrate the high degree of order of the prepared gold nanoparticle array, the pair distribution function g(*r*) was calculated by first locating the particle positions using the particle analysis function of NIH’s ImageJ [18], subsequently generating a histogram *N*(*r*) of the interparticle distances, and finally, applying the following equation for normalization:

(1) $$\text{g}\left(\text{r}\right)=\text{N}\left(\text{r}\right)/2\pi \text{r}\Delta \text{r}\rho$$

**Figure 3:**
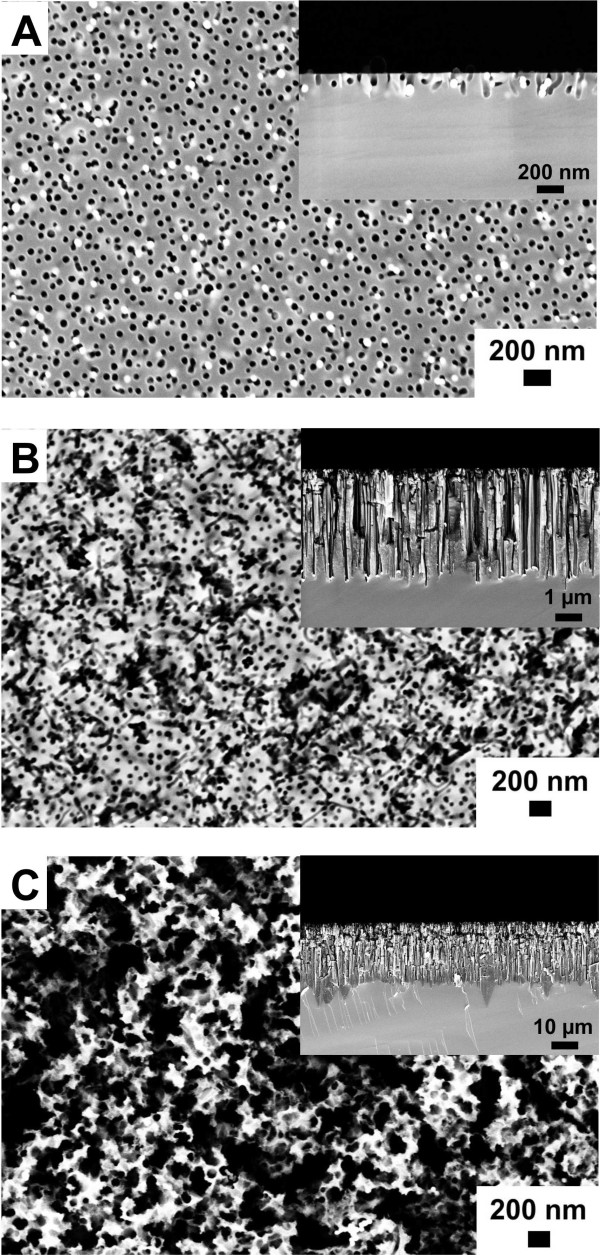
**SEM images of porous silicon etched for different time periods.** The Si substrate was immersed in piranha solution prior to spin-coating of the gold nanoparticles and was plasma-treated prior to metal-assisted etching. (**A**) Etching time, 1 min; (**B**) etching time, 10 min; and (**C**) etching time, 60 min. Insets show cross-sectional images of the corresponding samples.

**Figure 4:**
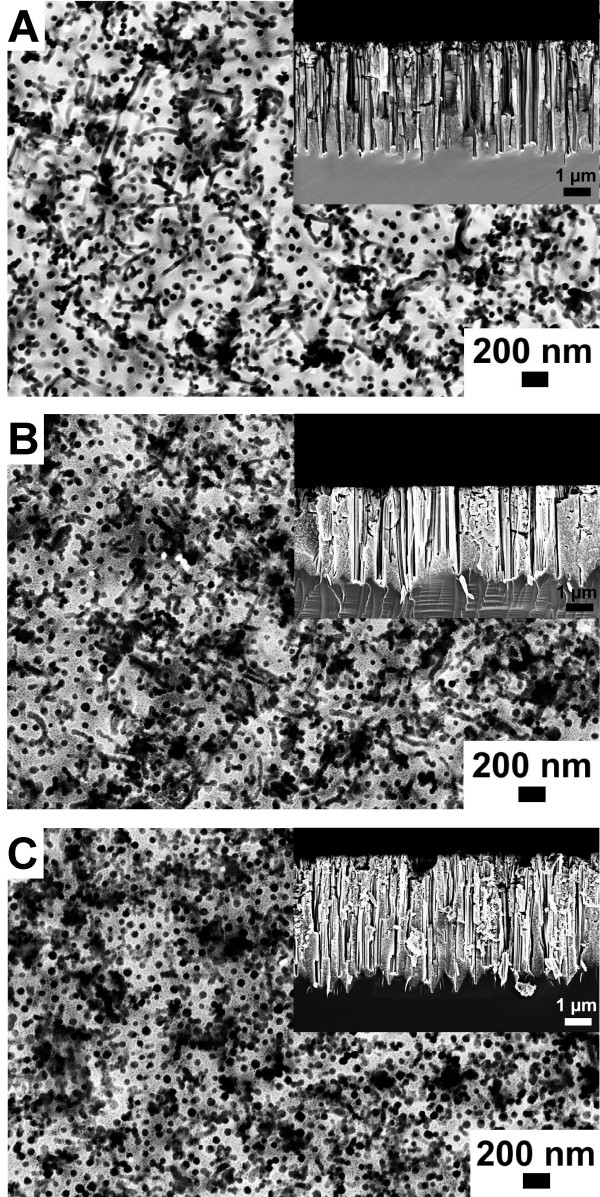
**SEM images of porous silicon prepared by metal-assisted etching using different fabrication strategies for gold nanoparticle arrays.** (**A**) Si substrate cleaned with piranha solution and polymer matrix removed by plasma treatment, (**B**) substrate cleaned with piranha solution and polymer matrix removed by flame annealing, and (**C**) substrate cleaned with HF and polymer matrix removed by flame annealing. Insets are corresponding cross-sectional SEM images.

Here, *N*(*r*) represents the number of particles in an annular disc of radii *r* and *r* + Δ*r* drawn with a particle at the center and *ρ* the number of particles per square nanometer. The pair distribution function corresponding to the SEM image displayed in Figure 2A is shown in Figure 2B as black solid line. The peak positions of the array coincide with the predicted peak positions for a perfect hexagonal lattice that are depicted in grey. From the curve we can extract the distance between the nearest particles as well as its variation, and we can obtain a rough estimate on the quality of the order by considering the visibility of the various peaks. Briefly, a higher degree of order or larger domain sizes result in a slower decay of the visibility of the peaks. The mean interparticle distance was slightly influenced by the surface properties of the Si substrate. Either the hydrophilic Si surfaces resulting from piranha treatment or the hydrophobic, H-terminated Si surfaces obtained by immersion in 4.8 M HF solution were used for the fabrication of highly ordered nanoparticle arrays. The interparticle distance of the nanoparticle arrays decreased from 111 ± 13 nm for the hydrophilic Si to 106 ± 12 nm for the hydrophobic Si. In addition the silicon surface properties had a strong impact on the subsequent plasma treatment step. The polymer matrix of the gold nanoparticles prepared on piranha-treated Si was completely removed in W10 (90% argon + 10% hydrogen) plasma after 45 min. In contrast, polymer residues were still present on HF-cleaned Si substrates after W10 (90% argon + 10% hydrogen) plasma treatment for 90 min. Consequently, these samples were not further processed. In order to investigate metal-assisted etching on Si substrates immersed in HF prior to spin-coating, another technique, referred to as flame annealing, was chosen for the removal of the polystyrene. Here, Si substrates decorated with polystyrene-coated gold nanoparticles were pulled through a propane/butane flame. The diameter of the gold nanoparticles was not significantly influenced by thermal treatment and was determined from analyzing SEM images to be 55 ± 9 nm. The key parameter of the fabricated gold nanoparticle arrays are presented in Table 1, and representative SEM images are shown in the Additional file 1. In summary for metal-assisted etching, three differently prepared gold nanoparticle arrays on Si were used which vary in the cleaning procedure of the Si substrates prior to spin-coating and/or in the thermal treatment method employed for the removal of the polymer matrix of the gold nanoparticles: (1) piranha treatment + plasma treatment, (2) piranha treatment + flame annealing, and (3) HF immersion + flame annealing. The first etching experiments were performed using gold nanoparticle arrays which were deposited on piranha-cleaned substrates and subsequently plasma-treated. The samples were immersed in a solution containing 4.8 M HF and 0.4 M H_2_O_2_ for different periods. SEM images of the resulting porous silicon layers are displayed in Figure 3. After 1 min etching time, most gold nanoparticles sank into the silicon (Figure 3A). However, the silicon etching rate was not reproducible in this case. Even on the same silicon substrate, gold nanoparticles could be found on top of the surface and burrowed in pores. An etching time of 10 min led to the formation of straight pores with a diameter of approximately 60 nm, reflecting the gold nanoparticle diameter (Figure 3B). In addition the pore density seemed to be correlated to the nanoparticle density before etching. The etching rate was determined to be 400 nm/min. Cross-sectional SEM images also show the generation of microporous silicon in between the larger pores. After etching for 60 min the pore openings are larger than the gold nanoparticle diameter indicating the dissolution of porous silicon pore walls close to the surface (Figure 3C). Figure 4 displays SEM images of porous silicon samples prepared by metal-assisted etching using different fabrication techniques for the generation of the gold nanoparticle mask. All samples were etched for 10 min. In Figure 4A a porous silicon sample is shown whose gold nanoparticle mask was deposited on piranha-cleaned silicon. In this case the polymer matrix was removed by plasma treatment. Obviously, the particle density and accordingly the pore density decreased significantly upon etching. Polymer matrix removal by flame annealing improved the pattern transfer as can been seen in Figure 4B,C. The ordered gold nanoparticle pattern was almost perfectly transferred into the silicon substrate (Figure 4B,C). This observation is supported by the appearance of rings in the fast Fourier transform (FFT) of the SEM images which show porous silicon originating from flame-annealed Si substrates (see Additional file 2). The surface properties of the Si substrate which result from piranha (hydrophilic) or HF treatment (hydrophobic) do not have a significant influence on the pattern transfer. However, we would like to emphasize that the gold nanoparticles did not sink homogenously into the silicon. Another major obstacle in the etching experiments is the formation of gas bubbles which interfere with the fabrication of homogenous porous silicon layers. Nevertheless, our results suggest that the fabrication method for the gold nanoparticle etching mask has an influence on the formation of porous silicon using metal-assisted etching.

**Table 1:** Key parameters of gold nanoparticle arrays prepared on silicon using different fabrication strategies

	**Diameter (nm)**	**Interparticle distance (nm)**
Piranha treatment	52 ± 9	111 ± 13
Piranha treatment + plasma treatment	55 ± 8	110 ± 12
Piranha treatment + flame annealing	56 ± 10	111 ± 18
HF treatment	56 ± 11	106 ±12
HF treatment + plasma treatment	-^a^	-^a^
HF treatment + flame annealing	54 ± 9	105 ± 15

^a^The diameter of the gold nanoparticles and their interparticle distances were not determined as the polymer matrix could not be completely removed with the chosen plasma conditions. Hence, the samples have not been used for metal-assisted etching.

## Conclusions

To summarize the porous silicon was prepared by metal-assisted etching of p-type silicon using highly ordered gold nanoparticle arrays as etching masks. Gold nanoparticles were wet-chemically synthesized and subsequently covered with polystyrene in order to enable the formation of highly ordered nanoparticle arrays on Si by self-assembly. The polymer shell of the gold nanoparticles was removed by either plasma treatment or flame annealing prior to metal-assisted etching. For the latter the gold nanoparticle-decorated Si substrates were immersed in a solution containing HF and H_2_O_2_. Depending on the fabrication process of the etching mask, the quasi-hexagonally, highly ordered array of nanoparticles was translated into a less-ordered array of pores. The best pattern transfer was achieved by using gold nanoparticle arrays whose polymer matrix was removed by flame annealing. The developed fabrication strategy can be extended to other wet-chemically synthesized metal nanoparticles and to the full potential of colloidal chemistry in order to study the influence of the size, shape, and material of the metal catalytic sites on the resulting porous silicon.

## Competing interests

The authors declare that they have no competing interests.

## Authors’ contributions

SPS synthesized polystyrene-coated gold nanoparticles. CP carried out all other experimental work including gold nanoparticle deposition, metal-assisted etching, and SEM characterization. In addition, CP designed the experiments and wrote the final version of the paper. SU determined the order of the gold nanoparticle arrays by analyzing SEM images and developed the method for transferring gold nanoparticles from the aqueous to the organic phase. SK conceived the method for the nanoparticle deposition. All authors read and approved the final manuscript.

## Supplementary Material

Additional file 1 **Low magnification SEM images of gold nanoparticle arrays fabricated using differently treated surfaces and annealing techniques.** SEM images of gold nanoparticle arrays prepared using differently treated surfaces and annealing techniques. (**a**) hydrophilic surface (piranha)/plasma treatment, (**b**) hydrophilic surface (piranha)/flame annealing, and (**c**) hydrophobic surface (HF treatment)/flame annealing. Scale bar is 1 μm. (DOC 4990 kb) Click here for file

Additional file 2 **Low magnification SEM images of a typical gold nanoparticle array and porous silicon samples fabricated by metal-assisted etching.** SEM images of a typical gold nanoparticle array and porous silicon samples which have been prepared by metal-assisted etching. The etching masks were fabricated using different methods. The insets show the corresponding FFTs. (**a**) gold nanoparticle array, (**b**) piranha solution/plasma treatment, (**c**) piranha solution/flame annealing, and (**d**) HF/flame annealing. (DOC 14557 kb) Click here for file
