# Supplementary material for: Fabrication of porous silicon by metal-assisted etching using highly ordered gold nanoparticle arrays
Source: Nanoscale Res Lett. 2012 Aug 9;7(1):450. doi: 10.1186/1556-276X-7-450 (PMC3463426; doi:10.1186/1556-276X-7-450)
Supplement: Additional file 1 — Low magnification SEM images of gold nanoparticle arrays fabricated using differently treated surfaces and annealing techniques. SEM images of gold nanoparticle arrays prepared using differently treated surfaces and annealing techniques. (a) hydrophilic surface (piranha)/plasma treatment, (b) hydrophilic surface (piranha)/flame annealing, and (c) hydrophobic surface (HF treatment)/flame annealing. Scale bar is 1 μm. (DOC 4990 kb) [file 1556-276X-7-450-S1.doc]

***Additional file 1***


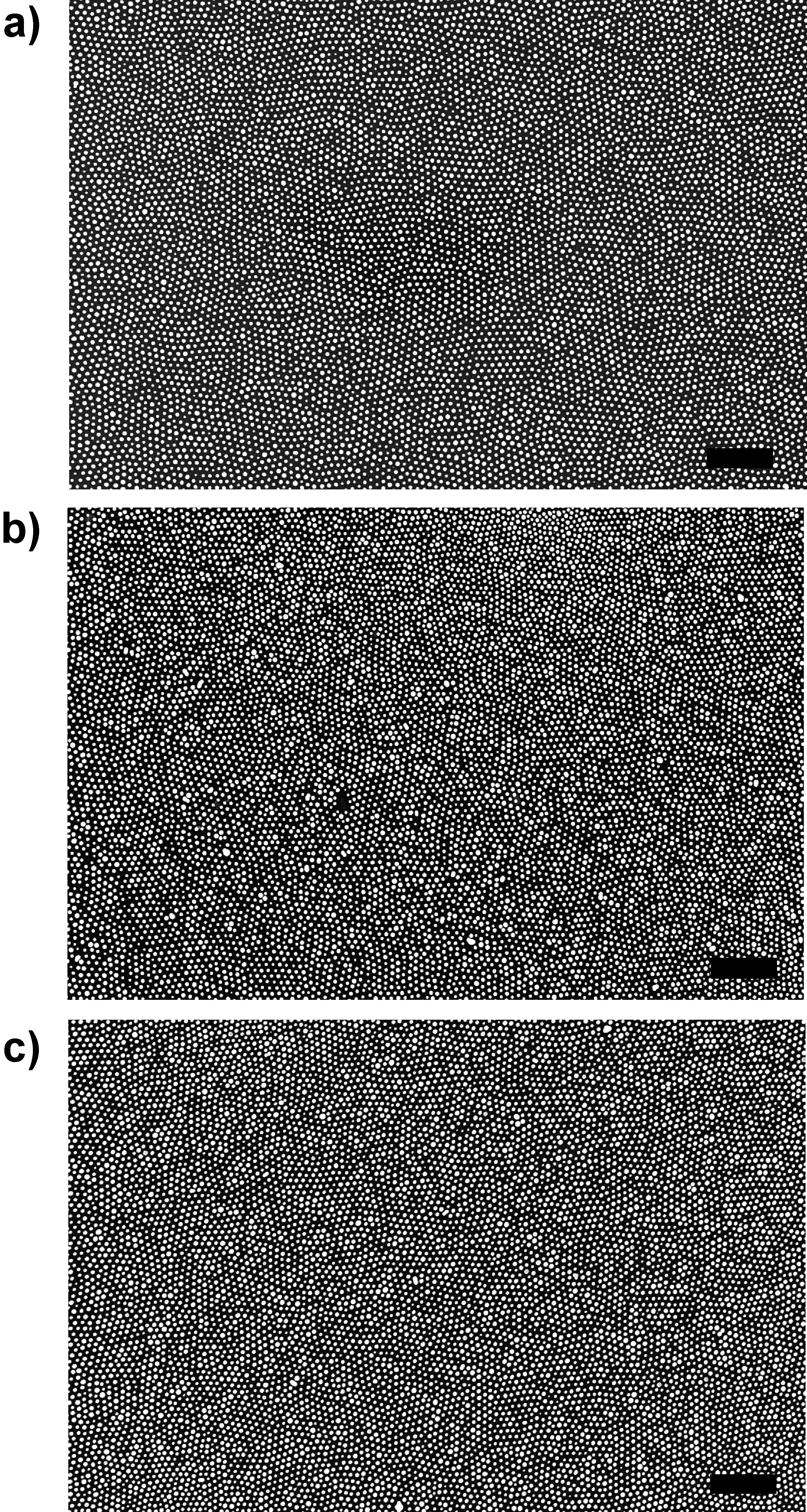


**Aditional file 1:** SEM images of gold nanoparticle arrays fabricated using differently treated surfaces and annealing techniques. a) hydrophilic surface (piranha)/plasma treatment, b) hydrophilic surface (piranha)/flame annealing, c) hydrophobic surface (HF treatment)/flame annealing. Scale bar is 1µm.
