# Supplementary material for: Fabrication of porous silicon by metal-assisted etching using highly ordered gold nanoparticle arrays
Source: Nanoscale Res Lett. 2012 Aug 9;7(1):450. doi: 10.1186/1556-276X-7-450 (PMC3463426; doi:10.1186/1556-276X-7-450)
Supplement: Additional file 2 — Low magnification SEM images of a typical gold nanoparticle array and porous silicon samples fabricated by metal-assisted etching. SEM images of a typical gold nanoparticle array and porous silicon samples which have been prepared by metal-assisted etching. The etching masks were fabricated using different methods. The insets show the corresponding FFTs. (a) gold nanoparticle array, (b) piranha solution/plasma treatment, (c) piranha solution/flame annealing, and (d) HF/flame annealing. (DOC 14557 kb) [file 1556-276X-7-450-S2.doc]

***Additional file 2***


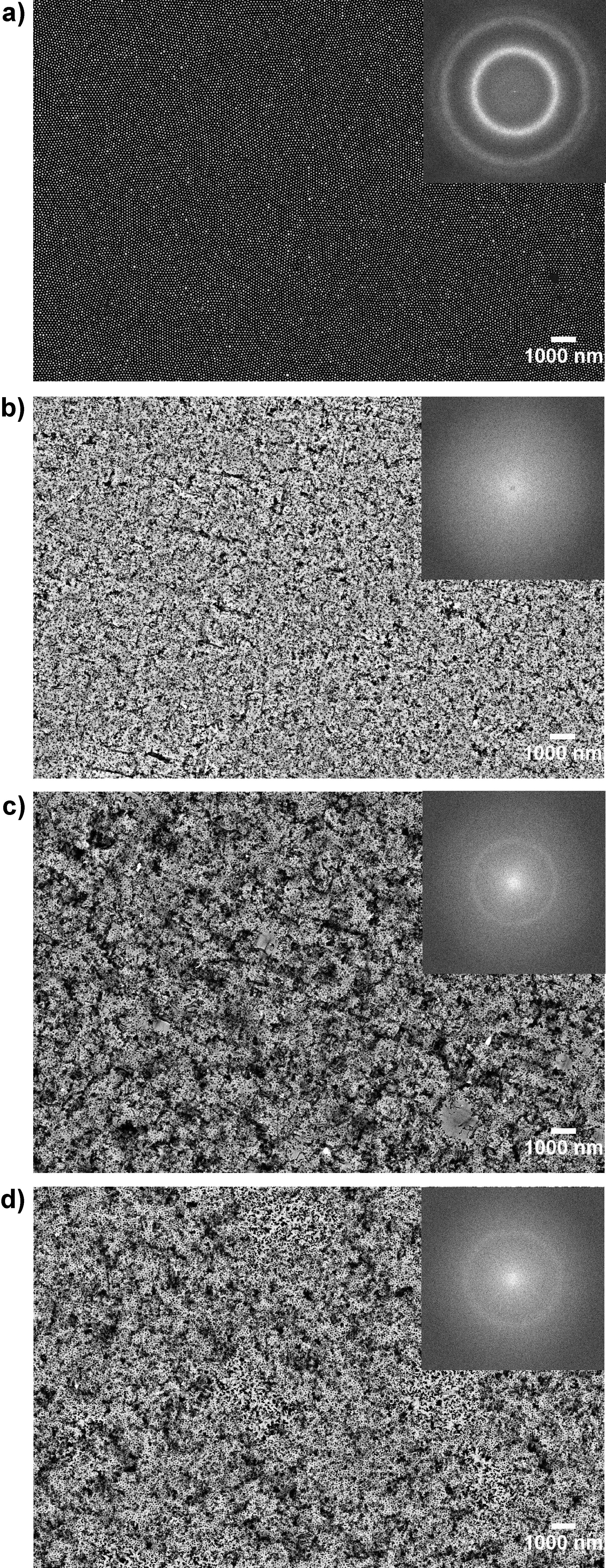


**Aditional file 2:** SEM images of a typical gold nanoparticle array and porous silicon samples which have been prepared by metal-assisted etching. The etching masks were fabricated using different methods. The insets show the corresponding FFTs. a) gold nanoparticle array, b) piranha solution/ plasma treatment, c) piranha solution/flame annealing, d) HF/flame annealing.
